# Differential effects of speech situations on mothers’ and fathers’ infant-directed and dog-directed speech: An acoustic analysis

**DOI:** 10.1038/s41598-017-13883-2

**Published:** 2017-10-23

**Authors:** Anna Gergely, Tamás Faragó, Ágoston Galambos, József Topál

**Affiliations:** 1 Institute of Cognitive Neuroscience and Psychology, Hungarian Academy of Sciences, Budapest, Hungary; 2 MTA-ELTE Comparative Ethology Research Group, Department of Ethology, Budapest, Hungary; 3 Eötvös Loránd University, Institute of Biology, Department of Ethology, Budapest, Hungary; 4 Eötvös Loránd University, Institute of Psychology, Cognitive Psychology Department, Budapest, Hungary

## Abstract

There is growing evidence that dog-directed and infant-directed speech have similar acoustic characteristics, like high overall pitch, wide pitch range, and attention-getting devices. However, it is still unclear whether dog- and infant-directed speech have gender or context-dependent acoustic features. In the present study, we collected comparable infant-, dog-, and adult directed speech samples (IDS, DDS, and ADS) in four different speech situations (Storytelling, Task solving, Teaching, and Fixed sentences situations); we obtained the samples from parents whose infants were younger than 30 months of age and also had pet dog at home. We found that ADS was different from IDS and DDS, independently of the speakers’ gender and the given situation. Higher overall pitch in DDS than in IDS during free situations was also found. Our results show that both parents hyperarticulate their vowels when talking to children but not when addressing dogs: this result is consistent with the goal of hyperspeech in language tutoring. Mothers, however, exaggerate their vowels for their infants under 18 months more than fathers do. Our findings suggest that IDS and DDS have context-dependent features and support the notion that people adapt their prosodic features to the acoustic preferences and emotional needs of their audience.

## Introduction

People tend to talk differently to babies and to pet dogs than they do to adults and the acoustic and linguistic features of such infant directed speech (IDS) and dog directed speech (DDS) proved to be very similar e.g.^1^ When compared to adult-directed talk, both infant- and dog-directed speech register have higher overall pitch (mean fundamental frequency – F0) and pitch variation (F0 range), lower mean length of utterance, and higher repetitiveness^2–5^. Striking similarities between dog- and infant-directed speech led to the hypothesis that the function of IDS is not limited to language tutoring, but plays the less specific role of engaging and maintaining the addressees’ attention, while eliciting their social responsiveness^1^. Although DDS and IDS share the aforementioned acoustic characteristics that have attentional and affective functions but only IDS has been characterized by vowel hyperarticulation (i.e. exaggerated acoustic information in the vowel formants), which appears to serve a didactic function and relates to the addressee’s linguistic competence^2,6,7^. Moreover, mothers even adjust their speech characteristics to the infants’ age and cognitive or linguistic abilities^8^. In contrast, a recent study suggests that the vowel changes in IDS are the result of vocal tract shortening (that can provide smaller body size information and being non-threatening) and not different articulation behaviour that can be linked to speech production and thus language tutoring^9^. These results suggest that people might unintentionally adapt their speaking style to the presumed linguistic skills, acoustic preferences, and emotional needs of their audience.

Importantly, however, in almost all experiments that aimed to compare IDS and DDS, talks were recorded only in one specific context (playing with the infant and/or the pet dog – see^2–4,6^), or data were obtained only from women’s spontaneous speech towards their dogs^1^. In a recent paper, Jeannin *et al*.^10^ studied how, during interactions with their dog, female owners adjust their speech characteristics based on the social context. They found that women tend to use elevated pitch when greeting their dogs after a separation, while low pitch and low pitch variance characterized their speech at the moment they were leaving the dog. However, the joint effect of gender and communication context is still unknown and this represents a clear limitation in the current literature because the audience’s needs may differ in various contexts and the speaker’s expectations about the addressee’s capacity to understand language can be situation- and gender-specific e.g.^3,11–13^

Existing evidence suggests that women and men differ in the use of verbal communication toward their dogs: the utterances used by women resemble more closely infant-directed speech^14^ and there are other gender-specific differences in acoustic features of DDS^15^. Namely, women tend to use infant directed speech register more than men while playing with a dog, and both genders’ talking style is more similar to IDS when interacting with an unfamiliar dog than when they interact with a familiar dog.

Moreover, comparative investigation of gender differences in infant directed speech (c.f. motherese and fatherese^16^) and adult directed speech (ADS) has shown that both mothers and fathers use heightened pitch in IDS compared to ADS while playing or during natural discourse (e.g. in storytelling situations)^12,17–21^. It is also worth noting that differences in the overall pitch can be language-specific. Fernald *et al*.^12^, for example, found that American English parents increased the mean-F0 in ID speech relative to AD speech significantly more than French, Italian, German, or British English parents did.

The analysis of pitch range in the aforementioned comparative studies^12,17,18,21^, however, resulted in a less coherent picture on how gender differences are manifested in IDS and ADS. One study showed that mothers (unlike fathers) used a wider pitch range while talking to infants aged between 0–24 months than when talking to adults^12^, while another study found that fathers increased their pitch range when talking to their 2-year-old toddlers even more than mothers did^18^. Finally, there are also other studies which found no gender-related differences in the use of pitch range during IDS versus ADS^17,21^.

Importantly, however, very few investigations have focused on the context-specificity of the acoustic features of IDS and most of these examined pitch contours rather than F0 mean or range. These studies show that rising contours are more frequent when the mother encourages her infant to make eye contact or to make a vocal turn e.g.^22^. Falling contours are more likely to occur while the mother is trying to comfort her crying infant e.g.^23^, whereas bell-shaped contours are usually used to reward the infant e.g.^24^. To our knowledge, there are only two studies that investigated mean-F0 and F0 range in two different situations and during IDS and ADS. These studies aimed to compare mothers’^20^ or fathers’^19^ speech while interacting with their children, aged 1 to 3 years, and with an adult experimenter, following a very similar design in both occasions. These interactions included both a reading aloud situation (i.e. reading a story book) and a free-speech situation (i.e. discussing with their partner a set of pictures depicting a park during different seasons). In line with other studies, in both situations, mothers used higher F0 mean and wider range in IDS (motherese) compared to ADS; these differences were significantly greater during the ‘free-speech’ than in the ‘reading aloud’ situation^20^. On the contrary, the comparison between ADS and IDS produced by fathers (fatherese) showed only minor differences: the only observed difference was that, in the free-speech situation, fatherese was characterized by significantly higher mean-F0 compared to the reading aloud situation^19^.

Based on the aforementioned studies, it is clear that the effects of gender and communication context on the acoustic characteristics of dog-directed speech require a more systematic (and comparative) investigation of dog- infant- and adult-directed speech samples. In the present study, therefore, we collected comparable samples from interactions with three different addressees (IDS, DDS and ADS) from both mothers and fathers who also had a pet dog at home. In order to investigate the effect of the speech situations on acoustic features of the different speech registers, participants were presented with three different ‘*Free-speech’* situations (Storytelling, Task solving and Teaching) and one ‘*Fixed sentences’* situation. ‘*Free-speech*’ situations were selected because each one closely resembles everyday parent-child interactions and such interactions rely heavily on prosodic features of speech. We chose the situations in order to collect data on the situation-specific nature of IDS, DDS and ADS. We may assume that the three ‘*Free-speech’* situations are different in terms of the speaker’s didactic intention and evoke different degrees of attention from the addressee (the Storytelling and the Task solving may require more attention eliciting features, while Teaching more language tutoring and didactic elements).

As the age of the infant can also modulate the attentional, affective, and didactic features of speech even within situations and conditions, we recruited families with infants covering wide range of speech and language development to test this effect. The age groups were selected to represent different levels of attentional and linguistic levels. The first two age categories we used (mean age ± SD: 4.8 ± 1.75 months and 16.5 ± 1.25 months) can be categorized as ‘pre-linguistic stage’ while children of the 25.5 ± 3.5 months group are in ‘emerging language stage’. The main difference between the two pre-linguistic groups is the different level of attentional skills and responsiveness which develop substantially after the 6th month of life^25^.

Whereas the inclusion of ‘*Fixed sentences*’ situation allowed us to obtain sound samples using a more standardised procedure, where we could rule out some of the partner-specific effects and other potential confounding factors that can differ when the adult interacts with an infant, toddler, dog or an adult partner.

## Results

### ‘Free speech’ situations – Mean fundamental frequency (F0)

**Figure 1:**
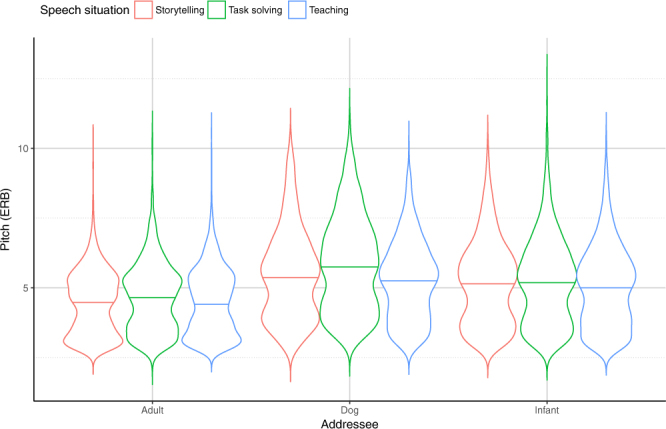
Mean of the fundamental frequency (F0) in mothers and fathers (pooled) when speaking to different partners in different ‘Free speech’ situations. Horizontal solid line indicates median, the width of the boxes indicate the distribution of the data points.

According to the model selection algorithm, the initial model including all possible two-way interactions was the most parsimonious (LR = 4621.773, p < 0.001; for details see Supplementary Table S1). We found a significant Addressee x Situation interaction in the speech samples: Pairwise comparisons (Supplementary Table S2) revealed that, in all of the three situations, both mothers and fathers used higher F0 in the DDS and in the IDS conditions than talking to an adult partner (ADS) (Fig. 1). Moreover, we found higher mean F0 values in DDS compared to IDS samples for both men and women (DDS > IDS > ADS).

The comparative analysis of the three ‘*Free speech*’ situations showed similar patterns in mothers’ and fathers’ pitch characteristics. Specifically, all situations differed significantly from each other: the highest mean F0 value was recorded in the *Task solving* situation, the lowest in *Teaching*, while *Storytelling* was in-between irrespective of the type of addressee (Task > Story > Teach). Both the Role*Addressee (Supplementary Table S3) and the Role*Situation (Supplementary Table S4) interactions showed that mothers used significantly higher pitch in general (Mother > Father).

Infants’ age significantly interacted with all fixed explanatory variables (Addressee, Situation, Role), as measured by the mean F0 values of the speakers. However we did not find a significant age effect within the speech situations, except for the *Task solving* situation, during which the parents of 4.8 ± 1.75 month-old infants used significantly higher pitch than the parents of 16.5 ± 1.25 month-old infants ([4.8 m > 16.5 m] = 25.5 m) (Supplementary Table S5). Within each age group, the mean F0 values show a similar pattern as the one described above: adults used significantly higher pitch in *Task solving* than in *Teaching* situation and *Storytelling* was between the two. The 25.5 ± 3.5 months old age group was an exception to this pattern, as *Teaching* and *Storytelling* did not differ significantly.

The age group proved to be a significant explanatory variable in IDS but not in DDS or ADS conditions (Supplementary Table S6). Mothers and fathers of the youngest infants (4.8 ± 1.75 months old) used the highest pitch; there was no difference between the pitches of the parents of the other two age groups (4.8 m > [16.5 m = 25.5 m]).

**Figure 2:**
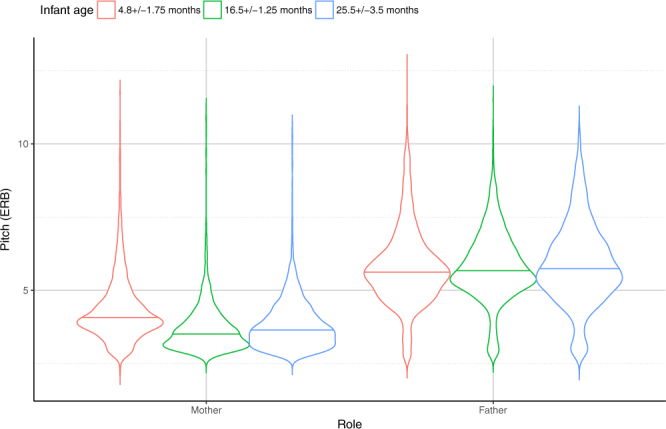
Mean of the fundamental frequency (F0) in mothers and fathers of the different age groups in the ‘Free speech’ situations. Horizontal solid line indicates median, the width of the boxes indicate the distribution of the data points.

Fathers adjusted their mean F0 to their children’s age: fathers of 4.8 ± 1.75 month-old infants used the highest pitch, while fathers with older children used significantly lower pitch during the interactions (4.8 m > [16.5 m = 25.5 m]). In contrast, we found no difference between age groups when looking at how mothers addressed their children (Fig. 2; Supplementary Table S7).

### ‘Free speech’ situations – Fundamental frequency (F0) range

In this analysis, the final model contained all two ways interaction effects of the factors except Role*Addressee (LR = 253.4518, p < 0.001; for details see Supplementary Table S8). The analysis of F0 range showed significant effects of Addressee and Situation, both of which had an interaction with the Role of the adult speaker (i.e. mother or father) (Supplementary Tables S9 and S10). In all task situations and with all addresees, the mothers’ speech had higher pitch range than the fathers’ (Mother > Father). Additionally, during the *Storytelling* and *Teaching* situations, both mothers and fathers used broader F0 range in the DDS and IDS conditions compared to the ADS condition. In contrast, both fathers and mothers used similar F0 range during dog- and infant-directed speech ([DDS = IDS] > ADS). In the *Task solving* situation, we found significant differences only between ADS and IDS conditions ([IDS > ADS] = DDS).

Pairwise comparisons of the three ‘*Free speech*’ situations show that mothers used broader pitch range during the *Task solving* compared to *Storytelling* and *Teaching*, and these last two situations did not differ from each other (Task > [Story = Teaching]). In contrast, fathers used wider F0 range during *Teaching* compared to *Storytelling*; while neither of the two situations differed from *Task solving* ([Teach > Story] = Task), irrespective of the type of addressee (infant/dog/adult).

Similarly to mean F0 values, infants’ age had a significant effect on fathers’ but not mothers’ pitch range (Supplementary Table S11). Fathers used significantly broader pitch range when speaking to 4.8 ± 1.75 month-old infants, while their speech samples were characterized by similar F0 range in the 16.5 ± 1.25 and in 25.5 ± 3.5 month-old toddler age groups (4.8 > [16.5 = 25.5]). Also, mothers’ and fathers’ pitch range differed only in the two older age groups (Mother > Father).

In the *Task solving* situation, parents of children in different age groups used similar pitch range (Supplementarty Table S12), while in both *Storytelling* and *Teaching* situations parents of 4.8 ± 1.75 month-old infants used significantly broader range than parents of 25.5 ± 3.5 month toddlers ([4.8 > 25.5] = 16.5). There was no effect of Situation within the youngest infant age group. In the 16.5 ± 1.25 month-old age group, both parents used significantly broader F0 range in the *Teaching* situation than in the other two situations (Teach > [Story = Task]). Parents of 25.5 ± 3.5 month-old infants, however, used significantly broader F0 range in the *Task solving* situation (Task > [Story = Teach]).

**Figure 3:**
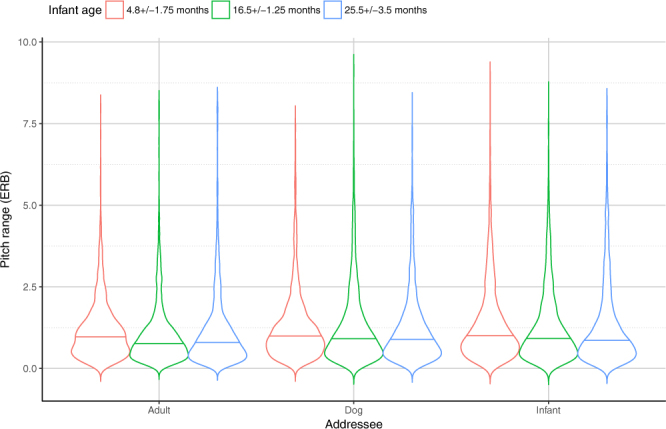
Fundamental frequency ranges in mothers and fathers (pooled) of the three age groups when speaking to different partners in the ‘Free speech’ situations. Horizontal solid line indicates median, the width of the boxes indicate the distribution of the data points.

Finally, in all age groups, mothers’ and fathers’ speech had significantly lower pitch range in the ADS condition compared to the other two addressees (Supplementary Table S13), while there were no significant differences between the DDS and the IDS conditions ([DDS = IDS] > ADS). Additionally, in the ADS condition, parents in the youngest infant age group used significantly higher F0 range than parents of older children (4.8 > [16.5 = 25.5]). There were no differences between age groups with the other addressees (Fig. 3).

### *‘Fixed sentences’* situation – Mean fundamental frequency (F0)

**Figure 4:**
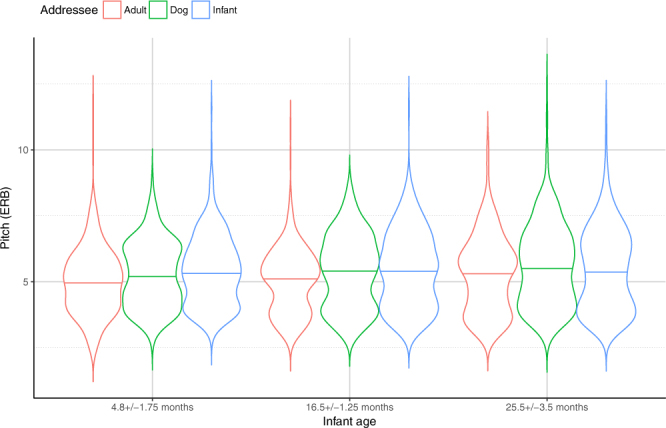
Mean of the fundamental frequency (F0) in mothers and fathers (pooled) of the three age groups when speaking to different partners in the ‘Fixed sentences situation. Horizontal solid line indicates median, the width of the boxes indicate the distribution of the data points.

In line with the results obtained from the analysis of *Free-speech* situations, we found that with all addressees mothers’ speech had significantly higher pitch than that of fathers (Mother > Father). There was also a significant Addressee*Age group interaction (LR = 264.0386, p < 0.001; for details see Supplementary Table S14). Infants’ age had no significant effect on parents’ mean F0 talking with the three addressees (IDS, DDS, ADS; Supplementary Table S15). However, parents of 4.8 ± 1.75 and 16.5 ± 1.25 month-old infants used significantly lower mean F0 in ADS than in DDS, and their mean F0 was significantly lower in the DDS than in the IDS condition (IDS > DDS > ADS; Fig. 4). In contrast to what found in the ‘*Free speech’* situations, in the oldest age group (25.5 ± 3.5 months), the mean F0 values of both mothers and fathers was similar when comparing speech to young children and to dogs ([DDS = IDS] > ADS).

### *‘Fixed sentences’* situation – Fundamental frequency (F0) range

**Figure 5:**
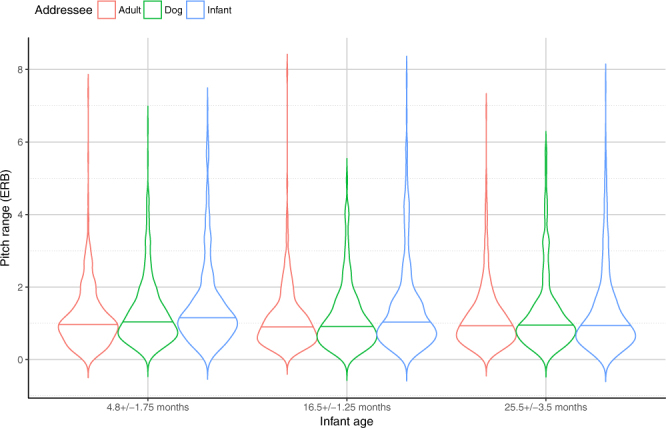
Fundamental frequency ranges in mothers and fathers (pooled) of the three age groups when speaking to different partners in the ‘Fixed sentences’ situation. Horizontal solid line indicates median, the width of the boxes indicate the distribution of the data points.

According to the final model (LR = 59.31615, p < 0.001; for details, see Supplementary Table S16), there was a difference in pitch range between mothers and fathers. Similarly to the *Free speech* situations, mothers’ voice was characterized by significantly broader range compared to fathers (Mother > Father); there was also a significant Addressee*Age group interaction in both mothers and fathers (Supplementary Table S17). We found a significant age effect only within the IDS condition: parents of 4.8 ± 1.75 month-old infants used a broader range than parents of 25.5 ± 3.5 month-olds, while 16.5 ± 1.25 month-olds did not significantly differ from neither of the other two age groups ([4.8 > 25.5] = 16.5). Parents of 16.5 ± 1.25 month-old infants used similar pitch range in ADS and DDS conditions, however their pitch range was significantly broader when they were talking to infants (IDS > [ADS = DDS]). In contrast, in the 4.8 ± 1.75 month-old age group parents used a significantly smaller pitch range in ADS condition as compared with DDS and IDS conditions ([DDS = IDS] > ADS). We found no effect of the addressee in the oldest age group (25.5 ± 3.5 months, Fig. 5).

### *‘Fixed sentences’* situation – Hyperarticulation

**Figure 6:**
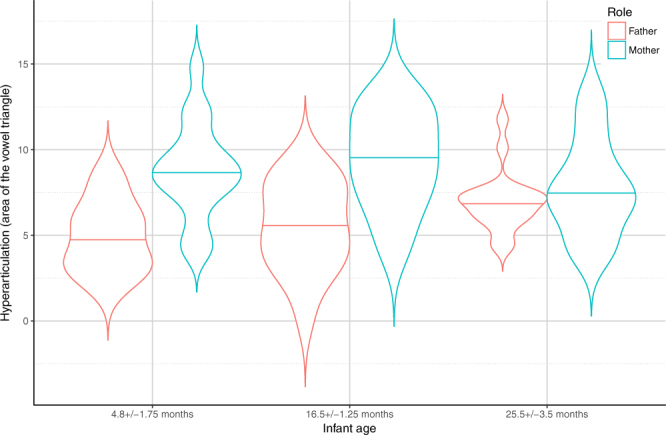
Hyperarticulation based on corner vowels in mothers and fathers in the three age groups. Horizontal solid line indicates median, the width of the boxes indicate the distribution of the data points.

In the final model (LR = 39.5136, p < 0.001; for details see Supplementary Table S18), we found that, in general, mothers hyperarticulated their vowels more than fathers (M > F), although hyperarticulation was also affected by Addressee (Supplementary Table S19). Both mothers and fathers hyperarticulated their vowels more when talking to their infants (IDS condition) as opposed to their dog (DDS) or the adult experimenter (ADS); both used similar articulation in the ADS and the DDS conditions (IDS > [DDS = ADS]). Finally, we found a differential effect of infants’ age on mothers’ and fathers’ hyperarticulation (Supplementary Table S20); mothers of 4.8 ± 1.75 and 16.5 ± 1.25 month-old infants used more articulated vowels than fathers, though there was no difference between male and female parents in the 25.5 ± 3.5 month-old age group (Fig. 6).

## Discussion

In the present study, we found evidence that both infant- and dog-directed speech have situation and gender dependent acoustic features that may change as a function of the emotional and attentional need of the addressee, as well as their language competence. Our results also suggest that prosodic features of people’s speech to their young children and dogs are more likely to be prominent in naturalistic social situations than during more controlled sentences, i.e. when addressors could interact with their partners only with certain restrictions.

The comparative analysis of the acoustic parameters of mothers’ and fathers’ speech samples obtained from interactions with different addressees provides further support to the notion that dog-directed talk shares many features with infant-directed speech. Moreover, it seems that both IDS and DDS markedly differ in their acoustic characteristics (i.e. heightened overall pitch and pitch range) from those of adult directed talk and these characteristics are displayed in a similar way in both men and women e.g.^15,21^. Contrary to previous findings^2,5,6^, our results showed that both mothers and fathers used higher pitch during DDS, compared to IDS. Importantly, however, this was the case only in the ‘*Free’* social interactions. In the *Fixed sentences* situation, the difference between DDS and IDS was only present in the 4.8 ± 1.75 and 16.5 ± 1.25 month-old age groups and disappeared in the parents who had a child 25.5 ± 3.5 months old. This result highlights some often-overlooked aspects of the experimental study of infant- and dog-directed speech, and raises the possibility that people talk to their partners differently in naturalistic, unrestricted situations as opposed to fully controlled situations in which participants are allowed to use only a predetermined set of phrases e.g.^2,26^.

Another possible explanation for the above differences in pitch characteristic is that the phenomenon is specific to Hungarian language. In fact, while the exaggerated acoustic characteristics of IDS are universal, e.g.^12^. language-specific variations have also been reported e.g.^27^. In line with this, we may assume that DDS might also have some language- and/or culture-specific features. Nevertheless, given the limited experimental data, the plausibility of this hypothesis is unclear at present.

Unlike mean pitch values, dog-directed speech was more similar to infant-directed talk in terms of pitch range. F0 ranges of both DDS and IDS differed from those used in ADS (although DDS did not differ from ADS in the *Task solving* situation). In this respect, it is also worth noting that the very same pattern was observed both in mothers’ and fathers’ speech samples. These results are consistent with the widely held notion that the two basic acoustic parameters of human speech (i.e. mean F0 and F0 range) probably play different roles; mean fundamental frequency is associated mainly with the communication of affection, while F0 range generally serves to draw the addressee’s attention e.g.^2,6,28^. Moreover, our results provide some support to the assumption that pitch and pitch range in IDS and DDS serve different functions, and that speakers utilize them differently according to the social situation and their partner’s needs e.g.^2,29,30^.

Concerning the context-specificity of DDS, IDS and ADS, we found that both women and men used a significantly higher pitch when they asked and encouraged their partner to complete an easy task (*Task solving*) compared to *Storytelling* or *Teaching* situations. However, the range of pitch they used when speaking to different partners showed different patterns between mothers and fathers. Men used the widest range during the *Teaching* situation, while women’s speech had the highest range in the *Task solving*. Furthermore, this effect was not independent from the interaction partner: men and women used similar range across the situations when talking to their dogs, they used significantly higher range in the *Task solving* (as compared with Teaching and Storytelling) when talking to an adult human, and used significantly higher range in both *Task solving* and *Teaching* (as compared with *Storytelling*) in the infant-directed speech condition.

Based on the finding that such changes in F0 and pitch range has the potential to facilitate attentional engagement of the addressee to the task^30^, we may conclude that this result is an indication that talkers adjust the acoustic parameters of their speech to their partners’ attentional and motivational state and further emphasise the potentially different roles of pitch and pitch variability. The latter seems to be more sensitively modulated by the task situation and the language comprehension abilities of the interaction partner, probably due to its attention-attracting function. Evidently, however, more (comparative) research is needed to better understand the relationship between contextual changes and varying characteristics of infant- and dog-directed talk.

Another intriguing question concerns the sex-specific differences in the acoustic parameters of dog- and infant-directed talks. Previous studies have shown that, according to independent raters, although women tend more frequently than men to use a ‘baby talk’-like speech register when playing with their dogs, both male and female owners use basically similar prosodic and lexical features during these interactions^4,15^. The acoustic features of DDS (i.e. mean fundamental frequency or pitch and pitch range) were not measured in these studies, rather, speech samples were only rated as using a baby-talk speech register or not. Our study provides more articulated acoustic evidence that women and men talk to their dogs and to their young children using similar speech register which is characterized by elevated pitch and pitch range and thus markedly differ from adult-directed talk. The finding that acoustic characteristics of speech samples in both men and women followed a similar pattern in terms of prosodic exaggeration (DDS > IDS > ADS) suggests that both mothers and fathers tend to express emotions with highly positive valence when communicating with their young children or dogs in ways that increase the addressees’ social responsiveness^28,30^.

The most striking difference between mothers and fathers was the age specificity of their baby talk speech register. While motherese seemed to be very similar toward infants in all our age categories, fatherese proved to be more sensitive to the infant’s age. More precisely, fathers used significantly higher pitch and broader pitch range when speaking to their 4.8 ± 1.75 moths-old babies than to older infants and toddlers. It is noteworthy that there are inconsistent findings among studies exploring the age-related changes in acoustic modifications of infant-directed speech. Although some studies have clearly demonstrated that motherese shows age-related changes in prosodic features during the first 24 months of an infants’ life^31–33^, others found no age-related changes in IDS^34,35^. The main reason for inconsistent results is that there is a wide variety of methods and protocols to assess the age-related changes in infant-directed speech c.f.^31^. As far as we know only one study investigated IDS of mothers and fathers previously with a comparative methodology in which free conversations were recorded from parents of 2- and 5 year-old children^18^. This study revealed that both sexes used elevated pitch and wider range toward 2-year-old toddlers, but only mothers used elevated F0 and wider range toward 5-year-olds compared to adult listeners while fathers’ voice was similar in this condition^18^. Our results further support the age-specific differences in motherese and fatherese: mothers used intense speech prosody independent of the infants’ age, while fathers show decreasing speech prosody toward older infants. It is possible that the ‘*Free*’ speech situation is the key to solve these contradictions, as in our study the ‘*Fixed*’ sentences showed no such age effect difference between mothers and fathers.

In addition, we found that both mothers and fathers hyperarticulated their vowels when talking to their infants, but not when addressing their dogs. This is in line with previous studies suggesting that hyperspeech is related to language tutoring e.g.^36–38^. Results from our study also indicate that mothers tend to hyperarticulate their vowels more when they have young infants (below 18 months) compared to fathers in all speech situations, while this difference disappears in the oldest age group. Other studies, however, reported that hyperarticulated vowels can be found not only in adult-children interactions but also when speaking to pets (cats and dogs^5^; dog puppies^39^) and computer avatars^40^. Based on these we may suppose that the tendency to provide a form of didactic support (language teaching) is just one of several possible factors that modulate vowel space expansion in IDS. For example, speech rate by itself can influence vowel hyperarticulation, thus the slower speech rate which occurs during infant- and dog-directed speech, can make all vowels to be more salient^4,41,42^. Positive facial expressions, like smiling, also influence the acoustic parameters of speech and has the potential to increase hyperarticulation of /i/ and /a/^43^ and others suggest that the degree of vowel hyperarticulation is strongly influenced by the feedback from the listener to the speaker^44,45^. However, recent findings of Kalashnikova *et al*.^9^ showed that the shortening of the vocal tract affects mainly the formant structure of the vowels in IDS suggesting a pre-linguistic function of hyperarticulation as manipulating the apparent body size of the speaker and communicating non-aggressive intent and social convergence.

It is important to note that our set of vowels in the ‘*Fixed sentences*’ was relatively limited maybe affecting the generalizability of our results. Another possible limitation of our study is that even with the best effort to keep our recording scenarios natural and familiar for participants, the use of the recording equipment, the presence of the unfamiliar experimenters and even using the experimenters as adult interaction partners could have an impact on the characteristics of the speech of participants and thus could introduce confounding in our sample. These procedural factors might be responsible for some of the differences between adult-directed and dog/infant-directed speech samples (for example, utterance length has been found to be different when talking to familiar and unfamiliar partner^15,46^).

In summary, we can conclude that our results support the hypothesis that IDS (motherese & fatherese) and DDS have context-specific features and these characteristics occur in both men’s and women’s speech. Dog-directed speech is characterized by more exaggerated speech prosody than infant-directed talk. Differences between IDS and DDS conditions suggest that infant-directed speech reflects a more flexible adjustment of speech characteristics (pitch, pitch range, hyperarticulation) to the presumed cognitive capacities of the addressee and to the social situation, and this may play an important role in creating and maintaining an efficient flow of information. Our results also shed light on the importance of comparative studies of mothers’ and fathers’ infant- and pet-directed speech considering not just the partner, but the age of the infant and the social situation of the interaction.

## Materials and Methods

### Ethics statement

This research was approved by the Human Research Ethics Committee (EPKEB) at Hungarian Academy of Sciences (No. 2015/23). In accordance with ethics approval, all parents completed an informed written consent to participate in the study and all methods were performed in accordance with the relevant guidelines and regulations of the EPKEB and the current laws of Hungary.

### Participants

A total of 21 families with 39 parents (21 women, mean age ± SD: 33.1 ± 5.6 years; 18 men, mean age ± SD: 37 ± 4.2 years, urban middle-class families, with high (60% of adults) to middle (40% of adults) education level) were recruited on a voluntary basis who had both biological child (younger than 30 months of age) and adult pet dog at home (dogs’ mean age ± SD: 5.3 ± 3.4 years). All participants spoke Hungarian as their native language except one father who spoke Dutch, therefore we excluded his recordings from the hyperarticulation analysis (see below in Procedure and Data analysis). One speech sample of one mother recorded during the Storytelling *“Free*” situation in the ADS condition was damaged (see below in Procedure and S1 Table).

In order to examine the effect of the infant’s age on motherese, families were grouped into three age categories: (1) parents of early pre-linguistic infants (N = 12, mean age 4.8 months ± 7 weeks); (2) parents of late pre-linguistic, infants (N = 12, mean age 16.5 months ± 5 weeks); (3) parents of infants in emerging language stage (N = 15, mean age 25.5 months ± 14 weeks). For IDS and DDS participants spoke to their own infant and dog. For ADS, they spoke to the adult female experimenter (in most of the cases GA [age: 29], on 6 occasions HKK [see acknowledgement, age:28]).

### Procedure

During the recording session two experimenters were present, one explained the tasks for the participants and play role in the interactions, while the other handled the recording equipment. Each mother and father was recorded speaking separately to their (i) infant (IDS), (ii) dog (DDS) and (iii) an adult experimenter (ADS) in a within-subjects design. Infant- dog- and adult-directed speech samples were collected from each participant in three *“Free*” situations (Storytelling, Task solving and Teaching) in which they were encouraged to speak freely without restrictions, and one “*Fixed*” situation where they were asked to say predefined sentences to the listeners (Fixed sentences) – see below for more details. In the ‘*Free’* situations our aim was not to get the Subjects to actually teach and transfer information, or to make the interaction partner to perform a task successfully, but to model situations that can be relevant and comparable across both species and the infants’ age groups.

#### Storytelling

Participants were instructed to call the listener’s (infant, dog or adult) attention to an object and tell a story about it. We used different target objects in the three different cases of addressees (a story book for IDS, a dog toy for DDS and a reproduction of a Van Gogh painting for ADS).

#### Task solving

Participants encouraged the listener to complete an easy task and praised them when they succeeded. In the IDS condition we used three different versions of the same object displacement task according to the infant’s age. The task for the parents of 4.8 ± 1.75 month-old infants was to encourage their baby to grab an object and give it to them. For the 16.5 ± 1.25 month-old infants the task was to displace ten similar objects from their initial place into a bag. The 25.5 ± 3.5 month-olds had to displace the same objects from left to right or vice-versa in a predetermined order with the constraints that the location of all objects needed to be changed. In the DDS condition the experimenter placed a tug, a rope and a tennis ball on the floor in front of the dog and then she named one of the objects. The participants’ task was to instruct the dog to select and retrieve the ‘named’ toy. In the ADS condition the same object displacement task was used as in the case of the 25.5 ± 3.5-month-old group.

#### Teaching

The procedure in this situation was identical for all addressees. Participants were instructed to explain how to use an optional application on their mobile phone to the listeners (i.e. which button must be pushed in order to open and run the application etc.).

#### Fixed sentences

Participants were asked to tell the following three sentences to the listeners (i.e. infant/dog/adult experimenter): (#1) *Nézd csak*, *milyen szép idő van odakint!* (in English: Just look! What nice weather!) (#2) *Akarsz sétálni egyet?* (in English: Do you want to go for a walk?) (#3) *Úgy látom*, *unatkozol*. *Nem csinálunk valami mást?* (in English: You seem really bored. Shouldn’t we do something else?).

Voice recordings were collected in a quiet room at the participants’ home using a Zoom H4n digital recorder with a head microphone (audio-technica, type: PRO 8HEx). Each recording lasted 30 to 60 seconds and participants were encouraged to interact with all listeners in a natural manner. To control for any potential order effects, speech recordings from the three addressees (IDS, DDS, ADS) were collected in a counterbalanced order across participants.

### Variables and Data Analysis

We analysed acoustic recordings from both ‘*Free’* and *‘Fixed’* situations with PRAAT software (version 6.0.05)^47^. First we annotated the recordings using a semiautomatic script in order to define and label pauses and calls and the exclusion of parts with background noise (instead of analysing utterances, we applied a call based approach for our analysis and defined call as one continuous production of a fundamental frequency).The baseline search range was defined between 75 Hz and 500 Hz, and before the pitch extraction the operator checked visually the detection of the pitch contour for halving and doubling errors, and modified the range if it was necessary. This way we could ensure the minimal level of artefacts in the measurements. Then we exported the following acoustic characteristics of each call from the program given on nonlinear scales to have the possibility of the comparison of mothers’ and fathers’ voice:^10^.

- Fundamental frequency (given on ERB scale): F0 (perceived as pitch) mean and range (calculated by subtracting F0 minimum from F0 maximum). Analysed using Praat’s built in cross correlation based Pitch extraction method.
- Vowel formants (given on bark scale): we also analysed first and second formants (F1 and F2) of the corner vowels (/i/, /u/, /a/) from phrase #3 (see above) of the *Fixed sentences* situation, in order to examine hyperarticulation. First each target vowel was labelled, average formant values were measured within vowels using Praat’s built in Formant function (extracted Formant number: 3, maximum Formant frequency: 3500), then plotted against F1 and F2, creating a triangle. Vowel triangle areas were then calculated for each of the three addressees (ADS, DDS, IDS) e.g.^2,6^.

All variables were analysed with random intercept general linear mixed-effect models (LMM) with Gaussian error distribution after box-cox transformation of the response to reach normal distribution and applying a nested random effect design (participant ID within family ID) in order to control for repeated measures and the lack of independence between mothers and fathers of the same family (see Supplementary Table S1), (R MASS package: boxcox function, nlme package, lme function). We applied automatic AIC based model selection in all LMM models to obtain the simplest models with the most explanatory power (MASS package step AIC function). These final models are reported in the Results (initial models are reported in the Supplementary Tables)

For the analyses of F0 mean and range in the *‘Free’* situations the fixed explanatory variables included Addressee (factor with 3 levels – IDS, DDS, ADS),Situation (factor with 3 levels – Task solving, Storytelling, Teaching), Role in the family (factor with two levels – mother, father), Age group of the infant (3-level factor – 4.8, 16.5, 25.5 months) and all possible two way interactions were added in the initial models In case of the *Fixed sentences* situation the initial model was the same as in the *Free situations* with the exclusion of Addressee factor (as there were no different interaction partners this time).

All tests were two-tailed and α value was set at 0.05. Tukey post-hoc test was applied for pairwise comparisons (lsmeans package, pairs function).

All data are available in the Supplementary Data.

## Electronic supplementary material

Supplementary Information

Supplementary Dataset
