# Supplementary material for: Differential effects of speech situations on mothers’ and fathers’ infant-directed and dog-directed speech: An acoustic analysis
Source: Sci Rep. 2017 Oct 23;7:13739. doi: 10.1038/s41598-017-13883-2 (PMC5653764; doi:10.1038/s41598-017-13883-2)
Supplement: Supplementary file 1 — Supplementary Information [file 41598_2017_13883_MOESM1_ESM.pdf]

Differential effects of speech Situations on mothers' and fathers' infant-directed and dog-directed speech: An acoustic analysis – Supplementary tables

Anna Gergely[1,a,*], Tamás Faragó[2,3,a] , Ágoston Galambos[1,4], and József Topál[1]

[1] Institute of Cognitive Neuroscience and Psychology, Hungarian Academy of Sciences, Budapest, Hungary

[2] MTA-ELTE Comparative Ethology Research Group, Department of Ethology, Budapest, Hungary

[3] Eötvös Loránd University, Institute of Biology, Department of Ethology, Budapest, Hungary

[4] Eötvös Loránd University, Institute of Psychology, Cognitive Psychology Department, Budapest, Hungary

[a] Contributed equally to the work

*Corresponding author: Anna Gergely, anna.gergely66@gmail.com, phone: +36 30 7577378

Free speech – Pitch

Table S 1

	Wald-test				Likelihood ratio test		
	numDF	denDF	F-value	p-value	LR	p-value	sign. Level
**(Intercept)**	1	29187	551256.2	<.0001			
**Situation**	2	29187	306.0	<.0001	568.4062	<.0001	***
**Role**	1	15	227.0	<.0001	68.76971	<.0001	***
**Addressee**	2	29187	1920.0	<.0001	3570.134	<.0001	***
**Age**	2	29187	2.6	0.0736	4.620502	0.0992	.
Situation:Role	*2*	*29187*	*36.5*	*<.0001*	*68.29963*	*<.0001*	***
Situation:Addressee	*4*	*29187*	*30.7*	*<.0001*	*115.501*	*<.0001*	***
Role:Addressee	*2*	*29187*	*47.2*	*<.0001*	*99.13085*	*<.0001*	***
Situation:Age	*4*	*29187*	*12.4*	*<.0001*	*47.90858*	*<.0001*	***
**Role:Age**	2	15	2.9	0.0865	5.030873	0.0808	.
Addressee:Age	*4*	*29187*	*31.1*	*<.0001*	*124.3262*	*<.0001*	***

Post-hoc tests:

Table S 2

**Situation**	**Addressee**	**estimate**	**SE**	**df**	**t.ratio**	**p.value**
	AD - DD	*0.013319310*	*0.0004380701*	*29188*	*30.405*	*<.0001*
Story (1)	***AD - ID***	*0.009060437*	*0.0004182895*	*29188*	*21.661*	*<.0001*
	DD - ID	*-0.004258873*	*0.0004291678*	*29188*	*-9.924*	*<.0001*
	AD - DD	*0.017365892*	*0.0004123034*	*29188*	*42.119*	*<.0001*
Task (2)	***AD - ID***	*0.009206016*	*0.0004055184*	*29188*	*22.702*	*<.0001*
	DD - ID	*-0.008159876*	*0.0004159493*	*29188*	*-19.617*	*<.0001*
	AD - DD	*0.011714378*	*0.0004035190*	*29188*	*29.031*	*<.0001*
Teaching (3)	***AD - ID***	*0.007837663*	*0.0004110813*	*29188*	*19.066*	*<.0001*
	DD - ID	*-0.003876715*	*0.0004296611*	*29188*	*-9.023*	*<.0001*
**Addressee**	**Situation**					
	1-2	*0.001954996*	*0.0004138934*	*29188*	*4.723*	*<.0001*
**AD**	***1-3***	*-0.001129879*	*0.0004049735*	*29188*	*-2.790*	*0.0146*
	2-3	*-0.003084875*	*0.0003929283*	*29188*	*-7.851*	*<.0001*
	1-2	*0.006001578*	*0.0004349676*	*29188*	*13.798*	*<.0001*
**DD**	***1-3***	*-0.002734811*	*0.0004358353*	*29188*	*-6.275*	*<.0001*
	2-3	*-0.008736389*	*0.0004229040*	*29188*	*-20.658*	*<.0001*
	1-2	*0.002100575*	*0.0004101188*	*29188*	*5.122*	*<.0001*
**ID**	***1-3***	*-0.002352652*	*0.0004232614*	*29188*	*-5.558*	*<.0001*
	2-3	*-0.004453228*	*0.0004232688*	*29188*	*-10.521*	*<.0001*

Table S 3

Addressee	Role	estimate	SE	df	t.ratio	p.value
AD	F - M	0.02986538	0.002202549	15	13.559	<.0001
DD	F - M	0.03459337	0.002206582	15	15.677	<.0001
ID	F - M	0.03293721	0.002204786	15	14.939	<.0001
**Role**	**Addressee**					
F	AD - DD	0.011769197	0.0003726444	29188	31.583	<.0001
	AD - ID	0.007165458	0.0003635582	29188	19.709	<.0001
	DD - ID	-0.004603739	0.0003838344	29188	-11.994	<.0001
M	AD - DD	0.016497189	0.0003140483	29188	52.531	<.0001
	AD - ID	0.010237286	0.0003130749	29188	32.699	<.0001
	DD - ID	-0.006259904	0.0003172580	29188	-19.731	<.0001

Table S 4

Situation	Role	estimate	SE	df	t.ratio	p.value
Story (1)	F - M	0.03266603	0.002205708	15	14.810	<.0001
Task (2)	F - M	0.03434641	0.002203665	15	15.586	<.0001
Teaching (3)	F - M	0.03038351	0.002204305	15	13.784	<.0001
**Role**	**Situation**					
F	1-2	0.0025121956	0.0003701608	29188	6.787	<.0001
	1-3	-0.0009311865	0.0003780379	29188	-2.463	0.0367
	2-3	-0.0034433821	0.0003690325	29188	-9.331	<.0001
M	1-2	0.0041925708	0.0003187356	29188	13.154	<.0001
	1-3	-0.0032137080	0.0003147235	29188	-10.211	<.0001
	2-3	-0.0074062788	0.0003111551	29188	-23.803	<.0001

Table S 5

Situation	Age	estimate	SE	df	t.ratio	p.value
Story (1)	1-2	-0.0058431518	0.002967753	15	-1.969	0.1543
	1-3	-0.0061530981	0.002774897	15	-2.217	0.1005
	2-3	-0.0003099463	0.002821349	15	-0.110	0.9934
Task (2)	1-2	-0.0081818864	0.002967268	15	-2.757	0.0369
	1-3	-0.0051924932	0.002774060	15	-1.872	0.1811
	2-3	0.0029893933	0.002816838	15	1.061	0.5515
Teaching (3)	1-2	-0.0051806454	0.002966533	15	-1.746	0.2211
	1-3	-0.0039806944	0.002773481	15	-1.435	0.3487
	2-3	0.0011999509	0.002817172	15	0.426	0.9055
**Age**	**Situation**	**estimate**	**SE**	**df**	**t.ratio**	**p.value**
4.8±1.75 (1)	1-2	0.0038117597	0.0004295236	29188	8.874	<.0001
	1-3	-0.0030174173	0.0004252653	29188	-7.095	<.0001
	2-3	-0.0068291770	0.0004365803	29188	-15.642	<.0001
16.5±1.25 (2)	1-2	0.0014730251	0.0004406767	29188	3.343	0.0024
	1-3	-0.0023549109	0.0004399039	29188	-5.353	<.0001
	2-3	-0.0038279360	0.0004266422	29188	-8.972	<.0001
25.5±3.5 (3)	1-2	0.0047723647	0.0003916581	29188	12.185	<.0001
	1-3	-0.0008450136	0.0003994862	29188	-2.115	0.0867
	2-3	-0.0056173783	0.0003805902	29188	-14.760	<.0001

Table S 6

Addressee	Age	estimate	SE	df	t.ratio	p.value
	1-2	-0.0058887537	0.002965806	15	-1.986	0.1500
AD	1-3	-0.0055147893	0.002771944	15	-1.990	0.1490
	2-3	0.0003739644	0.002815670	15	0.133	0.9903
	1-2	-0.0054169524	0.002968661	15	-1.825	0.1953
DD	1-3	-0.0017512277	0.002776080	15	-0.631	0.8057
	2-3	0.0036657247	0.002821109	15	1.299	0.4170
	1-2	*-0.0078999775*	*0.002967349*	*15*	*-2.662*	*0.0442*
ID	*1-3*	*-0.0080602687*	*0.002774624*	*15*	*-2.905*	*0.0277*
	2-3	-0.0001602912	0.002818633	15	-0.057	0.9982
**Age**	**Addressee**					
	AD - DD	*0.012721406*	*0.0004298651*	*29188*	*29.594*	*<.0001*
4.8±1.75 (1)	*AD - ID*	*0.010220273*	*0.0004303155*	*29188*	*23.751*	*<.0001*
	DD - ID	*-0.002501133*	*0.0004340605*	*29188*	*-5.762*	*<.0001*
	AD - DD	*0.013193207*	*0.0004357153*	*29188*	*30.279*	*<.0001*
16.5±1.25 (2)	*AD - ID*	*0.008209049*	*0.0004289567*	*29188*	*19.137*	*<.0001*
	DD - ID	*-0.004984158*	*0.0004444960*	*29188*	*-11.213*	*<.0001*
	AD - DD	*0.016484967*	*0.0003897484*	*29188*	*42.296*	*<.0001*
25.5±3.5 (3)	*AD - ID*	*0.007674793*	*0.0003800116*	*29188*	*20.196*	*<.0001*
	DD - ID	*-0.008810174*	*0.0004014505*	*29188*	*-21.946*	*<.0001*

Table S 7

Role	Age	estimate	SE	df	t.ratio	p.value
	1-2	*-0.0119795569*	*0.004198027*	*19*	*-2.854*	*0.0262*
F	*1-3*	*-0.0105229099*	*0.003781203*	*29188*	*-2.783*	*0.0149*
	2-3	0.0014566470	0.004064369	19	0.358	0.9319
	1-2	-0.0008242321	0.003880151	15	-0.212	0.9755
M	*1-3*	0.0003053861	0.003766500	15	0.081	0.9964
	2-3	0.0011296183	0.003609871	15	0.313	0.9476
**Age**	***Role***					
4.8±1.75 (1)	*F - M*	*0.02513744*	*0.003862066*	*15*	*6.509*	*<.0001*
16.5±1.25 (2)	*F - M*	*0.03629277*	*0.003965061*	*15*	*9.153*	*<.0001*
25.5±3.5 (3)	*F - M*	*0.03596574*	*0.003472016*	*15*	*10.359*	*<.0001*

Free speech – Pitch range

Table S 8

	Wald-test				Likelihood ratio test		
	**numDF**	**denDF**	**F-value**	**p-value**	**LR**	**p-value**	**sign. Level**
**(Intercept)**	1	29189	15574.071	<.0001			
**Situation**	2	29189	8.799	0.0002	18.83087	0.0001	***
**Role**	1	15	28.680	0.0001	19.26174	<.0001	***
**Addressee**	2	29189	57.137	<.0001	115.6264	<.0001	***
**Age**	2	29189	4.057	0.0173	6.38719	0.041	*
**Situation:Role**	2	29189	6.414	0.0016	14.01744	0.0009	***
**Situation:Addressee**	4	29189	4.797	0.0007	18.44862	0.001	**
**Situation:Age**	4	29189	11.670	<.0001	47.48617	*<.0001*	***
**Role:Age**	2	15	3.081	0.0757	5.459925	0.0652	.
**Addressee:Age**	4	29189	3.904	0.0036	15.62552	0.0036	**

Post-hoc tests:

Table S 9

**Situation**	**Role**	**estimate**	**SE**	**df**	**t.ratio**	**p.value**
1	***F - M***	*0.05219292*	*0.009973896*	*15*	*5.233*	*0.0001*
2	***F - M***	*0.05804782*	*0.009926859*	*15*	*5.848*	*<.0001*
3	***F - M***	*0.03993952*	*0.009938737*	*15*	*4.019*	*0.0011*
**Role**	**Situation**					
	1-2	0.005492205	0.003795202	29190	1.447	0.3168
F	***1-3***	*0.013745110*	*0.003872936*	*29190*	*3.549*	*0.0011*
	2-3	0.008252905	0.003780075	29190	2.183	0.0740
	1-2	*0.011347113*	*0.003267229*	*29190*	*3.473*	*0.0015*
M	***1-3***	0.001491719	0.003225445	29190	0.462	0.8888
	2-3	*-0.009855395*	*0.003190249*	*29190*	*-3.089*	*0.0057*

Table S 10

Situation	Addressee	estimate	SE	df	t.ratio	p.value
	AD - DD	*0.031910187*	*0.004472241*	*29190*	*7.135*	*<.0001*
Story (1)	***AD - ID***	*0.023312812*	*0.004274528*	*29190*	*5.454*	*<.0001*
	DD - ID	-0.008597375	0.004371650	29190	-1.967	0.1205
	AD - DD	0.009400806	0.004211430	29190	2.232	0.0659
Task (2)	***AD - ID***	*0.018210258*	*0.004147612*	*29190*	*4.391*	*<.0001*
	DD - ID	0.008809452	0.004251244	29190	2.072	0.0956
	AD - DD	*0.024106464*	*0.004107307*	*29190*	*5.869*	*<.0001*
Teaching (3)	***AD - ID***	*0.030417541*	*0.004189842*	*29190*	*7.260*	*<.0001*
	DD - ID	0.006311077	0.004365105	29190	1.446	0.3174
**Addressee**	**Situation**					
	1-2	*1.762364e-02*	*0.004243509*	*29190*	*4.153*	*0.0001*
**AD**	***1-3***	7.851412e-03	0.004152391	29190	1.891	0.1413
	2-3	*-9.772226e-03*	*0.004029180*	*29190*	*-2.425*	*0.0405*
	1-2	-4.885743e-03	0.004459477	29190	-1.096	0.5168
**DD**	***1-3***	4.768956e-05	0.004468668	29190	0.011	0.9999
	2-3	4.933433e-03	0.004334387	29190	1.138	0.4905
	1-2	*1.252108e-02*	*0.004203703*	*29190*	*2.979*	*0.0081*
**ID**	***1-3***	*1.495614e-02*	*0.004339452*	*29190*	*3.447*	*0.0016*
	2-3	2.435058e-03	0.004335469	29190	0.562	0.8405

Table S 11

Role	Age	estimate	SE	df	t.ratio	p.value
	1-2	*-0.054079932*	*0.01793524*	*19*	*-3.015*	*0.0186*
F	***1-3***	*-0.055636092*	*0.01642651*	*29190*	*-3.387*	*0.0020*
	2-3	-0.001556160	0.01735267	19	-0.090	0.9956
	1-2	-0.007495065	0.01643111	15	-0.456	0.8924
M	***1-3***	-0.003197035	0.01595594	15	-0.200	0.9781
	2-3	0.004298030	0.01529212	15	0.281	0.9575
**Age**	***Role***					
**4.8±1.75 (1)**	***F - M***	0.01705211	0.01693621	15	1.007	0.3300
**16.5±1.25 (2)**	***F - M***	*0.06363698*	*0.01722523*	*15*	*3.694*	*0.0022*
**25.5±3.5 (3)**	***F - M***	*0.06949117*	*0.01520438*	*15*	*4.570*	*0.0004*

Table S 12

Situation	Age	estimate	SE	df	t.ratio	p.value
Story (1)	**1-2**	-0.03307496	0.01278062	15	-2.588	0.0509
	**1-3**	*-0.03629777*	*0.01204657*	*15*	*-3.013*	*0.0224*
	**2-3**	-0.00322281	0.01220640	15	-0.264	0.9624
Task (2)	***1-2***	-0.03206909	0.01276798	15	-2.512	0.0587
	**1-3**	-0.01103549	0.01202413	15	-0.918	0.6378
	**2-3**	0.02103360	0.01209667	15	1.739	0.2237
Teaching (3)	**1-2**	-0.02721844	0.01275021	15	-2.135	0.1162
	**1-3**	*-0.04091642*	*0.01202536*	*15*	*-3.403*	*0.0103*
	**2-3**	-0.01369799	0.01211201	15	-1.131	0.5106
**Age**	**Situation**	estimate	SE	df	t.ratio	p.value
**4.8±1.75 (1)**	***1-2***	-0.0003363912	0.004403430	29190	-0.076	0.9968
	1-3	0.0072057881	0.004360458	29190	1.653	0.2238
	2-3	0.0075421793	0.004474271	29190	1.686	0.2107
**16.5±1.25 (2)**	***1-2***	0.0006694785	0.004518366	29190	0.148	0.9880
	1-3	*0.0130623157*	*0.004510596*	*29190*	*2.896*	*0.0106*
	2-3	*0.0123928372*	*0.004373963*	*29190*	*2.833*	*0.0128*
**25.5±3.5 (3)**	***1-2***	*0.0249258899*	*0.004015786*	*29190*	*6.207*	*<.0001*
	**1-3**	0.0025871388	0.004095973	29190	0.632	0.8027
	2-3	*-0.0223387511*	*0.003901865*	*29190*	*-5.725*	*<.0001*

Table S 13

Addressee	Age	estimate	SE	df	t.ratio	p.value
**AD**	**1-2**	*-0.043520678*	*0.01273204*	*15*	*-3.418*	*0.0100*
	**1-3**	*-0.032369867*	*0.01198754*	*15*	*-2.700*	*0.0411*
	**2-3**	0.011150811	0.01207390	15	0.924	0.6343
**DD**	**1-2**	-0.020640351	0.01280290	15	-1.612	0.2711
	**1-3**	-0.025527648	0.01207530	15	-2.114	0.1205
	**2-3**	-0.004887296	0.01220043	15	-0.401	0.9158
**ID**	***1-2***	-0.028201466	0.01276234	15	-2.210	0.1019
	1-3	-0.030352176	0.01203731	15	-2.522	0.0577
	**2-3**	-0.002150710	0.01213873	15	-0.177	0.9829
**Age**	**Addressee**					
**4.8±1.75 (1)**	***AD - DD***	*0.011898303*	*0.004391832*	*29190*	*2.709*	*0.0185*
	AD - ID	*0.018201236*	*0.004409384*	*29190*	*4.128*	*0.0001*
	DD - ID	0.006302933	0.004440517	29190	1.419	0.3308
**16.5±1.25 (2)**	***AD - DD***	*0.034778630*	*0.004432510*	*29190*	*7.846*	*<.0001*
	AD - ID	*0.033520448*	*0.004349410*	*29190*	*7.707*	*<.0001*
	DD - ID	-0.001258182	0.004495120	29190	-0.280	0.9577
**25.5±3.5 (3)**	***AD - DD***	*0.018740523*	*0.003980847*	*29190*	*4.708*	*<.0001*
	AD - ID	*0.020218927*	*0.003883294*	*29190*	*5.207*	*<.0001*
	DD - ID	0.001478404	0.004094372	29190	0.361	0.9307

Fixed sentences - Pitch

Table S 14

	Wald-test				Likelihood ratio test		
	numDF	**denDF**	**F-value**	**p-value**	**LR**	**p-value**	**sign. level**
**(Intercept)**	1	3598	628608.3	<.0001			
**Role**	1	18	234.7	<.0001	77.7416	<.0001	***
**Addressee**	2	3598	88.3	<.0001	172.34	<.0001	***
**Age**	2	18	0.1	0.9422	0.1185425	0.9425	
**Addressee:Age**	4	3598	3.5	0.0073	14.04073	0.0072	**

Post-hoc tests:

Table S 15

**Age**	**Addressee**	**estimate**	**SE**	**df**	**t.ratio**	**p.value**
**4.8±1.75 (1)**	***AD - DD***	*0.005930679*	*0.0009467641*	*3598*	*6.264*	*<.0001*
	AD - ID	*0.008113607*	*0.0009498589*	*3598*	*8.542*	*<.0001*
	DD - ID	*0.002182928*	*0.0009292436*	*3598*	*2.349*	*0.0494*
**16.5±1.25 (2)**	***AD - DD***	*0.004802284*	*0.0010768131*	*3598*	*4.460*	*<.0001*
	AD - ID	*0.007490741*	*0.0011074937*	*3598*	*6.764*	*<.0001*
	DD - ID	*0.002688457*	*0.0010613346*	*3598*	*2.533*	*0.0305*
**25.5±3.5 (3)**	***AD - DD***	*0.007236435*	*0.0009351710*	*3598*	*7.738*	*<.0001*
	AD - ID	*0.005373985*	*0.0009056570*	*3598*	*5.934*	*<.0001*
	DD - ID	-0.001862450	0.0009425488	3598	-1.976	0.1183
**Addressee**	**Age**					
**AD**	**1-2**	0.0003881801	0.002867746	18	0.135	0.9900
	**1-3**	0.0011701870	0.002704169	18	0.433	0.9025
	**2-3**	0.0007820068	0.002726338	18	0.287	0.9558
**DD**	**1-2**	-0.0007402145	0.002845183	18	-0.260	0.9635
	**1-3**	0.0024759427	0.002710386	18	0.914	0.6390
	**2-3**	0.0032161572	0.002722411	18	1.181	0.4789
**ID**	***1-2***	-0.0002346852	0.002855760	18	-0.082	0.9963
	1-3	-0.0015694348	0.002698922	18	-0.582	0.8316
	**2-3**	-0.0013347496	0.002721212	18	-0.490	0.8767

Fixed sentences – Pitch range

Table S 16

	Wald-test				Likelihood ratio test		
	numDF	denDF	F-value	p-value	LR	p-value	sign. Level
**(Intercept)**	1	3598	29136.861	<.0001			
**Role**	1	18	16.656	0.0007	15.22396	1,00E-04	***
**Addressee**	2	3598	15.684	<.0001	31.11536	<.0001	***
**Age**	2	18	1.849	0.1860	3.482143	0.1753	
**Addressee:Age**	4	3598	2.856	0.0223	11.43241	0.0221	*

Post-hoc tests:

Table S 17

**Age**	**Addressee**	**estimate**	**SE**	**df**	**t.ratio**	**p.value**
**4.8±1.75 (1)**	***AD - DD***	*-0.028883013*	*0.010062167*	*3598*	*-2.870*	*0.0115*
	AD - ID	*-0.048116142*	*0.010093201*	*3598*	*-4.767*	*<.0001*
	DD - ID	-0.019233129	0.009878236	3598	-1.947	0.1258
**16.5±1.25 (2)**	***AD - DD***	-0.002407778	0.011420457	3598	-0.211	0.9758
	AD - ID	*-0.041186752*	*0.011759095*	*3598*	*-3.503*	*0.0014*
	DD - ID	*-0.038778974*	*0.011268729*	*3598*	*-3.441*	*0.0017*
**25.5±3.5 (3)**	***AD - DD***	-0.017583461	0.009935393	3598	-1.770	0.1800
	AD - ID	-0.014855461	0.009615033	3598	-1.545	0.2700
	DD - ID	0.002728001	0.010016102	3598	0.272	0.9599
**Addressee**	**Age**					
**AD**	**1-2**	0.009983786	0.01751403	18	0.570	0.8376
	**1-3**	0.010204498	0.01622470	18	0.629	0.8063
	**2-3**	0.000220712	0.01677973	18	0.013	0.9999
**DD**	**1-2**	0.036459021	0.01708531	18	2.134	0.1109
	**1-3**	0.021504049	0.01634683	18	1.315	0.4051
	**2-3**	-0.014954972	0.01669985	18	-0.896	0.6499
**ID**	***1-2***	0.016913176	0.01732967	18	0.976	0.6009
	1-3	*0.043465179*	*0.01613280*	*18*	*2.694*	*0.0376*
	**2-3**	0.026552003	0.01672751	18	1.587	0.2764

Fixed sentences - Hyperarticulation

Table S 18

Hyperarticulation	Wald-test				Likelihood ratio test		
	numDF	denDF	F-value	p-value	LR	p-value	sign. Level
**(Intercept)**	1	71	617.5865	<.0001			
Role	1	14	21.5246	0.0004	15.292	1,00E-04	***
Addressee	*2*	*71*	*8.9532*	*0.0003*	*17.06478*	*2,00E-04*	***
**Age**	2	17	0.4619	0.6378	0.8641087	0.6492	
Role:Age	*2*	*14*	*3.0119*	*0.0817*	*5.979864*	*0.0503*	.

Post-hoc tests:

Table S 19

contrast	estimate	SE	df	t.ratio	p.value
**AD - DD**	-0.9027319	0.4611459	71	-1.958	0.1304
AD - ID	*-1.9957048*	*0.4611459*	*71*	*-4.328*	*0.0001*
DD - ID	*-1.0929730*	*0.4570870*	*71*	*-2.391*	*0.0503*

Table S 20

**Age**	***Role***	**estimate**	**SE**	**df**	**t.ratio**	**p.value**
**4.8±1.75 (1)**	***F - M***	*-3.7586876*	*1.0368376*	*14*	*-3.625*	*0.0028*
**16.5±1.25 (2)**	***F - M***	*-3.8633333*	*1.0791602*	*14*	*-3.580*	*0.0030*
**25.5±3.5 (3)**	***F - M***	-0.8188889	0.9624842	14	-0.851	0.4092
**Role**	**Age**					
	1-2	-0.5883333	1.0791602	17	-0.545	0.8503
F	***1-3***	-2.0561111	1.0289389	17	-1.998	0.1430
	2-3	-1.4677778	1.0791602	17	-1.360	0.3830
	1-2	-0.6929790	1.0368376	14	-0.668	0.7852
M	***1-3***	0.8836876	0.9709237	14	0.910	0.6430
	2-3	1.5766667	0.9624842	14	1.638	0.2629
